# Social Isolation: How Can the Effects on the Cholinergic System Be Isolated?

**DOI:** 10.3389/fphar.2021.716460

**Published:** 2021-11-30

**Authors:** Jaromir Myslivecek

**Affiliations:** Institute of Physiology, First Faculty of Medicine, Charles University, Prague, Czechia

**Keywords:** social stress, social isolation, nicotinic receptors, muscarinic receptors, cholinergic signaling, interactome

## Abstract

Social species form organizations that support individuals because the consequent social behaviors help these organisms survive. The isolation of these individuals may be a stressor. We reviewed the potential mechanisms of the effects of social isolation on cholinergic signaling and *vice versa* how changes in cholinergic signaling affect changes due to social isolation.There are two important problems regarding this topic. First, isolation schemes differ in their duration (1–165 days) and initiation (immediately after birth to adulthood). Second, there is an important problem that is generally not considered when studying the role of the cholinergic system in neurobehavioral correlates: muscarinic and nicotinic receptor subtypes do not differ sufficiently in their affinity for orthosteric site agonists and antagonists. Some potential cholinesterase inhibitors also affect other targets, such as receptors or other neurotransmitter systems. Therefore, the role of the cholinergic system in social isolation should be carefully considered, and multiple receptor systems may be involved in the central nervous system response, although some subtypes are involved in specific functions. To determine the role of a specific receptor subtype, the presence of a specific subtype in the central nervous system should be determined using search in knockout studies with the careful application of specific agonists/antagonists.

## Introduction

A number of species, including humans and other mammals, such as rodents, are social animals. However, all living species, –– such as plants (Murphy and Dudley, 2009) and fungi (Aleklett and Boddy, 2021), exhibit social behavior in some form. Social species form organizations that have evolved with behavioral, neural, hormonal, cellular, and genetic mechanisms. These mechanisms support individuals because the consequent social behaviors help these organisms survive, reproduce, and care for offspring (Cacioppo et al., 2011). Cohen and Syme (1985) structurally delineated social isolation in elderly individuals as the absence of social interactions, contacts, and relationships with family, friends, neighbors on an individual level, and “society at large” on a broader level (Cohen and Syme, 1985). However, social isolation is not age-dependent, and it affects individuals regardless of their age. Therefore, a similar definition may be applied to animals.

We propose the following definition of social isolation in animals: the absence of contacts and interactions with the members of one’s own group to which the individual has no antagonistic relationship.

We emphasize the absence of antagonistic relationships in this definition. Animals generally cooperate in a group, but if antagonism, primarily between males, develops, aggressive behavior generally occurs, which may induce similar changes in the central nervous system as social isolation.

Social isolation is a stressor (Myslivecek and Kvetnansky, 2006), and the increased activity of the catecholaminergic system and further release of glucocorticoids (Myslivecek, 2015) are tightly connected with the separation of social animals from the natural group. Social isolation increases the responses to beta-adrenergic stimulation in mice (Francès et al., 1980). Therefore, it is not surprising that social isolation also affects animal activity. Social isolation decreases motor activity (Frances et al., 1981)) and drug-evoked activity (Lapiz et al., 2003) and increases fighting behavior (Davanzo et al., 1966; Yoshimura and Ueki, 1977). Isolation-reared rats have significantly higher locomotor activity than group-reared rats when exposed to novel cages (Lapiz et al., 2003). Lapiz et al. (2003) reported that isolation-reared rats had reduced presynaptic noradrenergic function in the hippocampus, enhanced presynaptic dopaminergic (DA) function in the amygdala and enhanced presynaptic DA and serotonin (5-HT) function in the nucleus accumbens, which was associated with decreased presynaptic 5-HT function in the frontal cortex and hippocampus. These changes contributed to changes in aggressivity, as reported by Davanzo et al., 1966 and Yoshimura and Ueki (1977), who demonstrated cholinergic changes in these behaviors (see below for detail).

Social isolation changes the effects of numerous drugs. This review focuses on the role of the cholinergic system in social isolation. Notably, social isolation changes the hypothermic effect of the muscarinic agonist oxotremorine (Frances et al., 1981).

Other effects of social isolation include changes in learning (Petkov and Rousseva, 1984; Wongwitdecha and Marsden, 1996; Ouchi et al., 2013), anxiety (Cheeta et al., 2001), adaptation to a strong sensory stimulus (e.g., prepulse inhibition (Shao et al., 2014)), and communication, e.g., ultrasonic vocalization in rodents (Branchi et al., 2004).

All of these functions are connected with the cholinergic system, which is widely associated with learning and memory (see Wongwitdecha and Marsden, 1996 for initial consideration), including conditional fear memory (Okada et al., 2015). The acute administration of nicotine produces anxiolytic effects, and social isolation shifts the response to nicotine without changing locomotor activity (Cheeta et al., 2001). Prepulse inhibition is an adaptation to sensory stimuli. Isolation induces prepulse inhibition deficits, and these effects are mediated via muscarinic receptors (MRs) and acetylcholinesterase [e.g., (Koda et al., 2011)]. Ultrasonic vocalization is under cholinergic control. Oxotremorine and different salts of atropine exert diverse effects on ultrasonic vocalization [see (Branchi et al., 2004) for details].

Social deprivation also influences brain development (Lapiz et al., 2003), affecting the development of neurotransmitter function, which alters brain development and subsequent adult behavior (similar to certain psychiatric disorders schizophrenia and depression). Sensory deprivation may affect the development of cognitive neural pathways (Lapiz et al., 2003). Social isolation is a factor in neurodevelopmental disorders (Matsumoto et al., 2019), such as autism spectrum disorder and specific learning disabilities.

Social isolation has been suggested as an animal model of schizophrenia primarily because of the similarity between the attenuation of prepulse inhibition in isolated animals and schizophrenic patients (Leng et al., 2004). Some data indicate (Ouchi et al., 2013) that social isolation provides an epigenetic animal model of attention-deficit hyperactivity disorder (ADHD). Possible connections between social isolation and neuropsychiatric diseases have been reviewed previously. Matsumoto et al. (2019) stated that social isolation causes behavior changes, such as reduced attention, impaired social affiliation behavior, and impaired conditional fear memories. Neuropharmacological analyses have revealed that different neuronal mechanisms modulate these behavioral features, which suggests that social isolation in mice can be used to establish an animal model of comorbid symptoms of patients with developmental disorders, including ADHD, autism spectrum disorder, and specific learning disabilities.

Studies of the effects of social isolation use different isolation schemes (see Table 1). Table 1 illustrates the variability in applied social isolation schemes. However, the table does not represent the full view of schemes. The data in this table were derived from references that described the role of cholinergic signaling in the development of central nervous system reactions to social isolation. The animal is generally isolated until the experiments are performed. However, there are some gaps between near-continuous isolation when the animal is subjected to more tests (see Table 1). Isolation lasted from 1 h to 165 days (i.e., approximately 5.5 months), and the isolation started on different days of animal development (from birth to adulthood, see also Table 1). Therefore, one obvious problem is that the isolation length affects the adaptation of animals to this unusual situation. Similarly, the beginning of isolation at birth occurs during a critical developmental period, which may worsen the effects of isolation. However, we did not identify any study of the cholinergic system that compared long vs short and early vs late isolation. Therefore, the reader can use Table 1 for better orientation in interpreting the results of studies on the role of cholinergic signaling in social deprivation.

**TABLE 1:** The parameters of social isolation.

Start of isolation	Number of isolation episodes	Duration of isolation	Species	References
26–28 days	1 (3 days before drug study)	21 +3 days (aggressors)/1, 7, 14, 21 days	mouse	Davanzo et al. (1966)
birth	1	20, 30, 60 days	cat	Kling et al. (1969)
25^th^ day	1	165 days	mouse	Essman, (1971)
adulthood	1	30 days	rat	Yoshimura and Ueki, (1977)
4^th^ week	1	3 months	rat	Oehler et al. (1980)
weaning	1	6–7 weeks	mice	Frances et al. (1981)
3^rd^ day	1	3^rd^ - 14^th^ day for 16 h/day	rat	Plaschke et al. (1987)
weaning	1	4, 8 weeks	rat	Heritch et al. (1990)
weaning	1	6 weeks	rat	Wongwitdecha and Marsden, (1996)
weaning (day 28)	1	8 weeks	rat	(Cilia et al., 2001; Cilia et al., 2005)
2 months	1	2 weeks	mouse	Hao et al. (2001)
adulthood	1	7 days	rat	Cheeta et al. (2001)
weaning	1	30 days	rat	Del-Bel et al. (2002)
weaning	1	6 weeks	rat	Muchimapura et al. (2003)
weaning (day 30)	1	60 days	gerbil	Lehmann et al. (2004)
weaning (day 21)	1	1 year	rat	Leng et al. (2004)
days 33–48 or 65–80	1	1 h	rat	Mccormick et al. (2005)
3 weeks	1	more than 6 weeks	mouse	Koda et al. (2008)
adulthood	1	1 month	mouse	Millan et al. (2008)
12 weeks	1	16 weeks	mouse	Huang et al. (2011)
3 weeks	1	6 weeks	mouse	Koda et al. (2011)
adulthood	1	2 h	rat	(Deurveilher et al., 2013)^a^
3 months	1	12 weeks	rat	Garrido et al. (2013)
4 weeks	1	6 weeks	mouse	Ouchi et al. (2013)
adulthood (day 38)	1	13 days (until day 51)	rat	Shao et al. (2014)
4 weeks	1 (repeated between tests)	2 + 1 weeks	mouse	Okada et al. (2015)
		2 + 1 weeks +1 + 4 days		
not given	1	6 weeks	mouse	Higashino et al. (2016)
adulthood	1	4 weeks	prairie voles	Grippo et al. (2018)
adulthood	1 (repeated between tests)	4 weeks + 11 days	mouse	Manouze et al. (2019)
adulthood	1 (repeated between tests)	70 days (with tests in between)	rat	Manouze et al. (2019)

Please note that adult rats/mice differ in strain and weight (e.g., age) at the beginning of the experiment, which is not specifically given in this table. The table illustrates the variability in applied social isolation and does not represent a full view of isolation schemes (see text for details).

a Rats were sleep deprived.

Social isolation is related to changes in nicotinic and muscarinic cholinergic signaling.

Specific MR and nicotinic receptor (NR) subtypes exhibit amino acid sequence similarity in orthosteric binding. This similarity is primarily true for MRs. The orthosteric binding site does not differ sufficiently to enable the synthesis of subtype-specific ligands. Orthosteric agonists and antagonists are not subtype specific to one MR subtype, although the manufacturer claims specificity. This lack of specificity may be overcome with the use of allosteric MR ligands, but there are some issues that should be mentioned. Receptors that generally exhibit high specificity for allosteric ligands have low affinity for these ligands (Kruse et al., 2014). This obstacle may be overcome with the use of bitopic ligands, which bind to orthosteric and allosteric sites. However, allosteric and orthosteric ligands are not generally used in behavioral and functional studies, and most of these ligands are not commercially available. Allosteric ligands specific to MR subtypes are relatively new, and ligands with a long history of use likely exhibit multiple targets compared to new drugs because the older ligands are better investigated.

## Cholinergic Signaling

**FIGURE 1:**
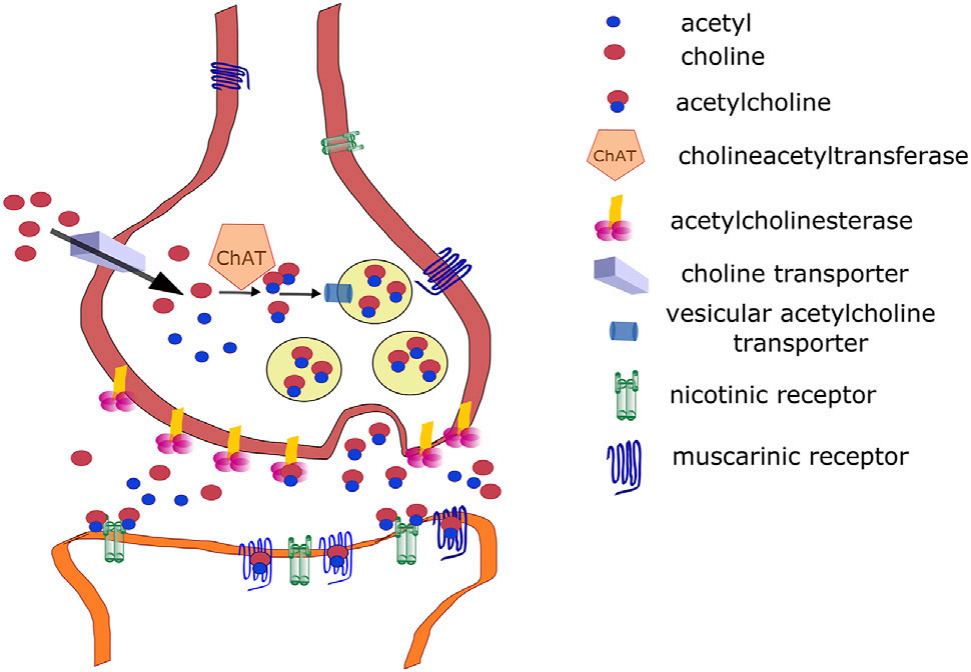
Cholinergic signaling. See legend for symbol explanation.

This section focuses on the role of the cholinergic system in the central nervous system (CNS) because social isolation primarily alters central responses. However, acetylcholine (ACh) plays an important role in autonomic nervous system physiology (Burnstock, 2009), including autonomic ganglia (Skok, 2002) and target parasympathetic neurons, which are cholinergic. Briefly, ACh is synthesized from choline and acetyl coenzyme A (Eiden, 1998). This reaction is catalyzed by choline acetyltransferase (ChAT). Choline is a product of ACh degradation via acetylcholinesterase (AChE), and it is taken up from the synaptic vicinity into presynaptic neurons by a high-affinity choline transporter (ChT) (Okuda and Haga, 2003) then into vesicles by a vesicular ACh transporter [VAChT, (Mallet et al., 1998)]. ACh release in the CNS is cleaved by AChE on the presynaptic membrane and anchored by a proline-rich membrane anchor (PRiMA) (Perrier et al., 2002). AChE is anchored by collagen Q (ColQ) in the periphery. Released ACh binds NRs (Dani, 2001) or MRs in the CNS (Brown, 2010). The synthetic pathways and target receptors are shown in Figure 1. For details on ACh synthesis and degradation, see the above-mentioned reviews.

MRs are typical members of the G-protein-coupled receptor (GPCR) family (Dencker et al., 2012; Myslivecek et al., 2017; Dean and Scarr, 2020). There are five subtypes of MRs, called M_1_-M_5_ (Brown, 2010; Thal et al., 2016). For simplification, class of receptors is divided into odd-numbered (M_1_, M_3_, M_5_) and even-numbered (M_2_ and M_4_) subtypes. Each of these “subfamilies” activate similar main intracellular signaling pathways (Brown, 2010). Odd-numbered MRs primarily couple to G_q_ proteins and activate the phospholipase C–inositol trisphosphate–diacylglycerol pathways), and even-numbered MRs are primarily coupled to G_i_ proteins, which inhibit adenylyl cyclase (Myslivecek et al., 2017). Other G proteins and ion channels may be activated, which constitute the next level of MR signaling complex (Tobin et al., 2009; Mighiu and Heximer, 2012; Myslivecek et al., 2017).

NRs are typical ion channel receptors that form hetero- or homopentamers composed of different subunits (Koukouli and Changeux, 2020). The structure (composition of subunits) of NRs divides these receptors into four groups, which were previously described according to tissue abundance. Neuronal NRs exist in two forms, bungarotoxin-sensitive (homopentameric receptor consisting of α_7-_α_9_ subunits) or bungarotoxin-insensitive receptors, which are heteropentameric and generally composed of two α_4_ subunits and three β_2_ subunits (Gotti and Clementi, 2004). However, receptors consisting of α_9_ subunits are found extraneuronally (Gotti and Clementi, 2004). Albuquerque et al. (2009)found the following types in brain slices: type IA, which consists of five α_7_ subunits; type II, which consists of α_4_β_2_ subunits; type III, which consists of α_3_β_4_β_2_ subunits; and type IV, which consists of α_2_β_4_/α_2_β_4_ subunits. NRs at the somatic neuromuscular junction of adult animals have the stoichiometry (α_1_)2β_1_δε (Alexander et al., 2017b). Autonomic ganglia express a combination of α_3_β_2_ or α_3_β_4_ (Skok, 2002). Therefore, huge variability in subunit composition exists. There is strong evidence that the pairwise assembly of some α and β subunits influences the biophysical and pharmacological properties of the receptor (Alexander et al., 2017b).

## Social Isolation and Cholinergic Mechanisms

Cholinergic mechanisms and their role in social isolation have been documented for decades. We divided cholinergic signaling into specific processes and reviewed connections to social isolation.

Neuropharmacological analyses revealed the role of many neurotransmitter systems in social isolation (Matsumoto et al., 2019). Increased motor activity is associated with the DA system, 5-HT system, and α-amino-3-hydroxy-5-methyl-4-isoxazolepropionic acid (AMPA) receptors and increased aggressiveness with many neurotransmitter systems, including noradrenergic, neurosteroid, and γ-aminobutyric acid (GABA)-ergic systems. Dysfunction of cholinergic systems is one of the underlying causes of fear memory deficits in socially isolated animals. Notably, social isolation affects neurotransmitter systems, but complex interplay between different neurotransmitter systems may occur.

However, the role of the cholinergic system is generally not considered in studies examining neurobehavioral correlates. MR subtypes do not differ sufficiently in their affinity to orthosteric antagonists (Farar and Myslivecek, 2016; Valuskova et al., 2018; Myslivecek, 2019) or orthosteric agonists (Alexander et al., 2017a). The nature of this phenomenon is the existence of amino acid sequence similarity in orthosteric binding sites in specific MR and NR subtypes (see also *Introduction*). NRs also have similar affinity to certain ligands (Alexander et al., 2017b). This affinity complicates the analysis of receptor subtypes and leads to some misinterpretation. Cholinergic drugs also have multiple effects. Tacrine is an AChE inhibitor and an allosteric muscarinic agonist (Wess, 2005), and galantamine allosterically affects NRs (Maelicke and Albuquerque, 2000).

Multiple ligands activate GPCRs (Vass et al., 2018). For example, 1312 potentially active ligands exist for M_1_ MRs, of which 930 are active at other GPCRs. Similarly, (Vass et al., 2018), among 1336 potentially active ligands for M_2,_ 974 ligands were active for other GPCRs. The corresponding numbers of ligands for M_3_ MRs and M_4_ MRs were 1384 vs 855 and 466 vs 387, respectively. Therefore, these authors discussed an interactome.

For example, the typical muscarinic antagonist atropine antagonizes the activation of the α_5_ subunit of NRs (Tomizawa and Yamamoto, 1992) and blocks the effects of drugs on α_2A_- (Carr et al., 2018) and α_1D_-adrenoceptors (Gaulton et al., 2016), glycine receptors (all subtypes, (Maksay et al., 1999; Yang et al., 2007)), solute carrier family 22 member 1 (SLC22a1, (Ahlin et al., 2008)), and serotonin 5-HT_1A_ receptors (Zhuang et al., 1993) and 5-HT_2C_ receptors (Gaulton et al., 2016) and inhibits AChE in mice (Sowell et al., 1992). Therefore, when atropine is used to block MRs, some side effects on α-adrenoceptors and 5-HT receptors may occur. Possible inhibition of AChE may artificially diminish the effects of MR blockade.

The effects of drugs on specific cholinergic mechanisms are shown in Table 2.

**TABLE 2:** The effects of drugs on specific cholinergic mechanisms.

Drug	Activity	Muscarinic receptors					Nicotinic receptors				Cholinesterases (ChEs)		Other targets
		M_1_	M_2_	M_3_	M_4_	M_5_	α_2_	α_4_	α_5_	α_7_	AChE	BuChE	
**Endogenous ligand**													
Acetylcholine	agonist	4.3–4.9	6.4	5.6	4.5–5.6	6.1	7.96	4–8.77		4.06–6.3	—		—
**Preferentially muscarinic**													
Carbachol	agonist	3.2–5.3	4.2–5.7	4.0–4.4	4.3–4.9	4.9	—	6.12		4.18	not cleaved by ChEs		—
Pilocarpine	agonist	4.9–5.1	4.9	5.1	5.2	5.0	—	—	—	—	—	—	—
Oxotremorine	agonist	5.5–6.0	5.0–6.6	5.3	5.2	5.1–7.26	—	—	—	—	5.82–8.77	—	—
Oxotremorine-M	agonist	5.1–5.6	4.9	5.1	5.2	—	—	—	—	—	—	—	—
Arecoline	agonist	5.7	5.2	5.4	5.5	—	6.65	6.57	6.65	—	—	—	CACNA1C
Bethanechol	agonist	4.0	4.0	4.2	4.0	—	—	—	—	—	—	—	—
Pirenzepine	antagonist	**7.8–8.5**	6.3–6.7	6.7–7.1	**7.1–8.1**	6.2–7.1	—	—	—	—	—	—	—
Atropine	antagonist	8.5–9.6	9.0–9.1	8.9–9.8	8.7–9.9	9.3–9.7	—	—	4.49	—	9.15–9.46	—	α_1D,2A_-AR, 5-HT_1A,2C_, SLC22A1, glycine
Benzatropine	antagonist	9.0	8.6	8.89–9.57	8.62–9.48	8.84–8.69	—	—	—	—	—	—	D_3_DR, α_1A,B,D_-AR α_2A,B,C_-AR,DAT, H_1_R, H_2_R, NET, 5-HT_2A,B,C,_ 5-HT_6_,SERT,σOR
Scopolamine	antagonist	9.0	8.7	9.4	9.5	—	—	—	—	—	—	—	—
Methylscopolamine	antagonist	9.9	—	10.4	—	—	—	—	—	—	—	—	—
N-desmethylclozapine	allosteric modulator	6.8–7.3	—	—	—	—	—	—	—	—	—	—	5-HT_2_, 5-HT_6_, 5-HT_7_
**Preferentially nicotinic**													—
Nicotine	agonist	—	—	—	—	—	7.92	6.89–8.66	—	4.04–4.87	—	—	TRPA1, K_v_4.3,5-HT_3_, σOR, SLC22A1, SLC22A2
Methyllycaconitine	antagonist	—	—	—	—	—	—	—	—	8.72	—	—	—
Mecamylamine	channel blocker	—	—	—	—	—	—	5.3–6.5	—	4.8	—	—	other α subunits (α_1,_ α_3,_ α_6_)
**Preferentially cholinesterase inhibitors**													
Rivastigmine	—	—	—	—	—	—	—	—	—	—	5.4	**7.4**	SERT
Physostigmine	—	4.43	5.87^a^				5.54^b^				7.6–7.8	7.6–7.8	CYP2D6
Pyridostigmine	—	6.09					5.70^c^				6.4	6.1	—
Galantamine	—	5.05					—	5.0	—	6.05	6.3	4.55–6.72	NMDAε2
Donepezil	—	6.08					4.52				7.7–8.3	4.82–7.7	ACAT, BACE 1, ChAc, H_3_R, MAO B, σ1, σOR
Tacrine	—	5.7 (allosteric)	5.7 (allosteric)	E	—	—	5.05–5.4^b^				7.5	7.2	ACAT, α_1A_-AR, β-amyloid A4 protein, CB1R, CB2R, ChAc, CYP2D6, DAT, GluN1, GluN2, MAO A, B, HNMT, NET, SERT, SLC22A1

The numbers show pKi. Only selected subunits are shown for NRs. Specificity (i.e., higher affinity than to other subtypes) is shown by a bold character. Please note that higher affinity means higher pKi. If the pKi was not given or could be computed, then the effects are shown as “E”. Unavailable data are shown as blank spaces. Data are from or adapted from (Pascuzzo et al., 1984; Cantí et al., 1998; Becerra et al., 2001; Lockhart et al., 2001; Samochocki et al., 2003; Harada et al., 2010; Alexander et al., 2017a; Alexander et al., 2017b; Myslivecek, 2019) and the IUPHAR/BPS Guide to Pharmacology (www.guidetophamacology.org). The activity represents the main effect of a specific ligand.

a Data available for all muscarinic receptor subtypes without specification.

b Data from electric eel.

c Data from muscle (frog, rat). CANCA1C, voltage-dependent L-type calcium channel subunit alpha-1C, subunit, α_1_-AR, α_1_-adrenoceptor; α_2_-AR, α_2_-adrenoceptor; 5-HT, serotonin receptors; SLC22A1; SLC22A, solute carrier 22; type 1, 2, glycine, glycine receptors; D_3_DR, dopamine D3 receptors; DAT, dopamine transporter; H_1_R, H_2_R, H_3_R, histamine receptors 1, 2, and 3; NET, norepinephrine transporter; TRPA1, transient receptor potential ankyrin channel 1; K_v_4.3, potassium channel; voltage-dependent, type 4.3; σOR, sigma opioid receptor; SERT, serotonin (5-HT) transporter; NMDAε2, ε2 subunit of NMDA; receptor; ACAT, acyl coenzyme A, cholesterol acyltransferase, BACE, 1, beta secretase 1; ChAc, choline acetylase; MAO A, B, monoamine oxidase A; B, σ1, σ1 receptor; CB1R, cannabinoid CB1, CB2 receptor; CYP2D6, cytochrome P450 2D6, GluN1; GluN2, glutamate NMDA, receptor (subunits 1 and 2); HNMT, histamine N-methyltransferase.

### Cholinergic Mechanisms: Acetylcholine Synthesis

ACh synthesizing enzyme activity (i.e., ChAT) differ in specific brain areas and species. No change was found in mice, but differences were found in rats. A decrease in ChAT activity was found in rats. These decreases were found with an increase in monoamine oxidase (MAO) activity in cortical regions (unfortunately homogenized together: forebrain, temporal, parietal, and occipital cortices) and subcortical regions (homogenized together: hippocampus, globus pallidus, thalamus, nucleus basalis of Meynert, caudate nucleus, putamen, and striatum) (Egashira, 2000). Other authors (Jones et al., 1991) found an increase in ChAT activity in the nucleus accumbens of socially isolated female rats.

No changes were found in ChAT activity (Hao et al., 2001) in mouse hippocampus. No change was found in the number of high-affinity ChTs. Social isolation did not affect ChAT activity in mouse prefrontal cortex or hippocampus (Koda et al., 2011).

Similar to ChAT activity, the data on ACh levels differ in specific brain regions. Neonatal (maternal) deprivation decreased the ACh concentration in whole infantile rat brain, which was prevented by neonatal administration of the AChE inhibitor pyridostigmine (Dörner et al., 1982). The ACh content was increased in the diencephalon of mouse-killing rats after social isolation (Yoshimura and Ueki, 1977). Briefly, individually housed rats were tested for mouse-killing behavior. Rats that exhibited the killing response were termed killers, and rats that failed to respond were termed nonkillers. The nonkiller rats had similar ACh levels to rats reared in the group. Isolation increases aggressive behavior. However, no change in the prefrontal cortex in response to acute restraint stress was observed following social isolation compared to enriched conditions (Garrido et al., 2013).

Isolation rearing *per se* changed cholinergic fiber densities in the prefrontal cortex of the left hemisphere and the entorhinal cortex of the right hemisphere (Lehmann et al., 2004) in gerbils. Early methamphetamine (on postnatal day 14) diminished the cholinergic innervation of the forelimb area of the cortex in both hemispheres in isolated gerbils and the left hemisphere in enriched-environment-reared gerbils and reduced cholinergic innervation in the hindlimb area on both sides in both rearing groups. These animals also had a poorer learning ability (special learning) and spent more time in horizontal activity in the open field between 10 and 20 min.

### Cholinergic Mechanisms: Acetylcholine Degradation

AChE activity was not changed due to social isolation. Therefore, no differences were found in regional AChE activity in cats reared in social isolation (Kling et al., 1969). AChE activity was not changed in rat cortex, striatum, amygdala, diencephalon or brainstem (Yoshimura and Ueki, 1977). No changes were shown in a subsequent study in these regions or the hypothalamus, midbrain, hippocampus, olfactory bulb, or pons plus medulla oblongata (Yoshimura, 1980). Hao and colleagues found no changes in AChE activity in the hippocampus (Hao et al., 2001). Social isolation did not affect AChE activity in mouse prefrontal cortex or hippocampus (Koda et al., 2011).

However, (Egashira, 2000), found decreased AChE activity in cortical regions (forebrain, temporal, parietal, and occipital cortices) and subcortical regions (hippocampus, globus pallidus, thalamus, nucleus basalis of Meynert, caudate nucleus, putamen, and striatum) in rats. Unfortunately, this author combined these brain areas.

Another aspect of ACh degradation is the inhibition of AChE. The inhibition of AChE increased the amount of ACh in the vicinity of neurons releasing ACh. However, there may be other off-target sites of AChE inhibitors (see Table 2). Neonatal treatment with pyridostigmine (an AChE inhibitor) between postnatal days 1 and 14 reduced the numbers of hippocampal CA1 and striatal synapses in isolated rats compared to treatment with placebo, and these numbers were diminished in isolated rats (Plaschke et al., 1986). However, the number of synapses was increased in isolated and isolated + pyridostigmine-treated rats at the age of 6 months. Other morphological changes were also found (Plaschke et al., 1987). The effects of galantamine (a weak competitive and reversible cholinesterase inhibitor that also acts as a potent allosteric ligand of human NRs α_4_β_2_, α_3_β_4_, and α_6_β_4_ and mouse nAChRs α_7_/5-HT_3_ (Samochocki et al., 2003) and acts on MRs (Lockhart et al., 2001)) and donepezil (a cholinesterase inhibitor that interacts with both MRs and NRs) on prepulse inhibition deficits elicited by isolation rearing were studied by Koda et al. (2008). Galantamine, but not donepezil, attenuated prepulse inhibition deficits, which may favor the nicotinic effects of galantamine (the affinity of NRs to donepezil is lower than galantamine) for AChE inhibition. However, the NR antagonists mecamylamine and methyllycaconitine did not prevent the galantamine-induced improvements in apomorphine and MK-801 prepulse inhibition deficits. Taken together, these findings show that complex interactions between MR and NR systems in social deficit-induced effects on prepulse inhibition may occur.

Higashino et al. (2016) found that rivastigmine (a cholinesterase inhibitor that inhibits AChE and butyrylcholinesterase) improved prepulse inhibition deficits.

Social isolation impaired prepulse inhibition but had no effect on spatial learning. Galantamine (an AChE inhibitor and allosteric ligand of nicotinic ACh receptors, see above) improved prepulse inhibition (Shao et al., 2014).

Tacrine (an AChE inhibitor and allosteric muscarinic agonist) had no effect on social affiliation deficits induced by social isolation (Okada et al., 2015).

### Cholinergic Mechanisms: Receptor Changes/Receptor Signaling

Cholinergic drugs very often affect nicotinic and muscarinic pathways. Therefore, we first discuss the effects on both signaling systems, then specifically describe the effects on nicotinic and muscarinic systems.

Social isolation-induced fighting behavior is suppressed by cholinergic (and many other) drugs, such as atropine (acting at NRs and MRs and AChE, see Table 2), scopolamine (acting at MRs), benzatropine (acting at MRs), and mecamylamine (acting at NRs (Davanzo et al., 1966)). The greatest effect was observed for the muscarinic antagonist scopolamine. Rats kept in isolation showed increased sensitivity of striatal neurons to ACh and dopamine (Oehler et al., 1980).

Tritiated ACh binds MRs and NRs. The main disadvantage of this ligand is its agonistic nature. Therefore, the release from binding sites is faster than that of an antagonist. The general difference between agonists and antagonists is that agonists exhibit fast association and fast dissociation and antagonists exhibit fast association and slow dissociation (Silverman, 2004). Tritiated ACh binding was similar in isolated and group-reared mice (Defeudis, 1972). However, these results do not indicate that NRs/MRs are unchanged because one receptor type may be increased while the other receptor is decreased.

#### Changes in Nicotinic Receptors/Signaling

Social isolation of mice for 165 days impaired passive and active avoidance, and nicotine reversed this effect (Essman, 1971). Nicotine oppositely reduced active avoidance in group-housed animals.

Nicotine attenuated the frequency of separation-induced distress vocalizations in chicks, and scopolamine increased these vocalizations. Pretreatment with the muscarinic antagonist scopolamine reduced the attenuating effects of nicotine (Sahley et al., 1981).

Herbut and Roliński (1985) examined the effects of cholinomimetics on aggression. Arecoline (a muscarinic and nicotinic agonist), carbachol (a muscarinic and nicotinic agonist), and physostigmine (an AChE inhibitor) potentiated isolation-induced aggression in mice, and cholinolytics (the muscarinic antagonists atropine and scopolamine) suppressed aggressive behavior. Cholinolytics with no central action (the example of that drug is ipratropium) did not inhibit aggression.

The cholinergic system is not the only system affected by social isolation, and some data showed an interaction between cholinergic and other neurotransmitter systems in socially deprived animals. Social isolation enhanced DA and 5-HT function in the nucleus accumbens (Lapiz et al., 2003), and it was associated with decreased presynaptic 5-HT function in the frontal cortex and hippocampus. Isolation-reared rats had reduced presynaptic noradrenergic function in the hippocampus but enhanced presynaptic DA function in the amygdala. These authors (Muchimapura et al., 2003) also studied the effect of social isolation on postsynaptic 5-HT_1A_ function in CA1 hippocampal neurons. The effects of the muscarinic (and nicotinic) agonist carbachol were similar in isolated and group-reared rats. Carbachol increased the firing rate of all neurons, and only a portion of these neurons showed a dose-response curve. Isolation rearing increased the sensitivity of neurons, which showed a concentration-related increase in firing in response to carbachol, but had no effect on the other neurons (Muchimapura et al., 2003).

Rats reared in social isolation postweaning have a deficit in auditory gating (O'neill et al., 2003). Auditory gating is a neurophysiological phenomenon in which within paired identical auditory click stimuli, the evoked potential to the second click is diminished compared to the first click. The ability to inhibit the response to the second stimulus is impaired in schizophrenic humans and rats reared in social isolation. The nicotinic α_7_ agonist 3-(2,4)-dimethoxybenzylidene anabaseine normalized impaired auditory gating.

One study (Bockman et al., 2018) compared NR properties and signaling in two groups, enriched-condition-reared and impaired-condition-reared rats. These authors found that the enriched condition-reared rats were less sensitive to the discriminative stimulus effects of nicotine. Mecamylamine (a nicotinic antagonist) inhibited the nicotine stimulus more potently in enriched-condition-reared rats. Epibatidine binding to NRs in the ventral tegmental area of enriched-condition-reared rats was reduced, and there was no difference in the nucleus accumbens.

Animals deprived of social contact show increased nicotine-induced locomotion (Noschang et al., 2021).

The effects of galantamine and donepezil (see also above) on prepulse inhibition deficits elicited by isolation rearing were studied by Koda et al. (2008). Galantamine, but not donepezil, attenuated prepulse inhibition deficits. Nicotinic ACh receptor antagonists did not prevent galantamine-induced improvements in prepulse inhibition deficits, which supports a complex interaction between MR and NR systems (see also above).

Social isolation (1 week or more) induced more spatial attention deficits in the water-finding test (Ouchi et al., 2013) with no sex difference and impaired contextual and conditional fear memory in the fear-conditioning test. Drugs used for ADHD therapy (methylphenidate or caffeine) improved social isolation-induced latent learning deficits in a manner that was reversible with mecamylamine (nicotinic) but not sulpiride (dopaminergic, SCH23390) antagonists.

#### Changes in Muscarinic Receptors/Signaling

The brain muscarinic system initiates aggression, and the nicotinic system inhibits aggressive behavior (Bell et al., 1985).

The muscarinic agonist oxotremorine produces hypothermia (Farar et al., 2012). The effect of salbutamol (a β_2_-adrenoceptor agonist) in oxotremorine-induced hypothermia was greater in isolated mice than group-reared mice (Francès et al., 1980). These data show that mutual interconnection occurs between muscarinic and β-adrenergic receptors. However, the number and affinity of β-adrenergic receptors in whole mouse brain (excluding the cerebellum) was not modified by isolation (Frances et al., 1981).

Nicotine attenuated the frequency of separation-induced distress vocalizations in chicks, and scopolamine (muscarinic antagonist) increased these vocalizations (Sahley et al., 1981, also see above).

Isolated male gerbils exhibited a higher frequency of scent marking than males reared in groups (Yoshimura, 1980). The muscarinic antagonist scopolamine suppressed marking behaviors, but the muscarinic antagonist methylscopolamine was ineffective.

Arecoline (a muscarinic and nicotinic agonist), bethanechol (a pure muscarinic agonist), carbachol (a muscarinic and nicotinic agonist), oxotremorine, pilocarpine (muscarinic agonists), and physostigmine (an AChE inhibitor with muscarinic and nicotinic effects) potentiated isolation-induced aggression in mice, and atropine (muscarinic and nicotinic antagonist) and scopolamine (muscarinic antagonist) suppressed aggressive behavior. Cholinolytics with no central action did not inhibit aggression (Herbut and Roliński, 1985).

Impaired learning (place learning and reversal learning) is a consequence of isolation rearing (Wongwitdecha and Marsden, 1996). Pretreatment with scopolamine (MR antagonist) produced a dose-related cognitive deficit, as shown by an increase in escape latency. Scopolamine impaired place and reversal learning, which was less pronounced in isolated rats. These results suggest that the interaction between learning and isolation is based on the MR system.

An increase in MR affinity and a decrease in the number of MRs in isolated rats (Egashira, 2000) were reported in cortical regions (forebrain temporal, parietal, and occipital cortices) and subcortical regions (hippocampus, globus pallidus, thalamus, nucleus basalis of Meynert, caudate nucleus, putamen, and striatum). No changes were observed in β-adrenergic receptors. Separation stress (Hao et al., 2001) increased hippocampal M_1_ MR mRNA and MR density (measured using pirenzepine, which is erroneously considered an M_1_ MR-specific ligand because this ligand also binds M_4_ MRs (see Table 2). An increase in α_2_-adrenoceptors was also found. However, other researchers found no changes in hippocampal M_1_ mRNA expression (Del-Bel et al., 2002), but they found an increase in 5-HT_1A_ mRNA expression in the dentate gyrus and CA3 areas of isolated animals.

Social isolation enhanced DA and 5-HT function in the nucleus accumbens (Lapiz et al., 2003) associated with decreased presynaptic 5-HT function in the frontal cortex and hippocampus. Isolation-reared rats have reduced presynaptic noradrenergic function in the hippocampus but enhanced presynaptic DA function in the amygdala. These authors further studied (Muchimapura et al., 2003) the effect of social isolation on postsynaptic 5-HT_1A_ function in CA1 hippocampal neurons. The effects of carbachol (muscarinic and nicotinic agonists) were similar in isolated and group-reared rats. Carbachol increased the firing rate of all neurons, with only a portion showing a dose-response curve. Isolation rearing increased the sensitivity of neurons, which showed a concentration-related increase in firing in response to carbachol but no effect on other neurons (Muchimapura et al., 2003). Therefore, complex interactions between the DA, 5-HT and cholinergic systems may be assumed, or the role of the interactome (Vass et al., 2018) should be considered.

Rodents use different means of communication, such as whistling, scent marking and ultrasonic vocalization, which are inaudible to humans. Ultrasonic vocalization is associated with the muscarinic cholinergic system (Wang et al., 2008). Social isolation affects ultrasonic vocalization. The muscarinic antagonist scopolamine increased the number of ultrasonic vocalizations (Branchi et al., 2004) during short isolation.

Another report found that social isolation led to only moderate changes in the DA system in the medial prefrontal cortex, but the cholinergic system was not affected (Leng et al., 2004).

Galantamine attenuated prepulse inhibition deficits (see also above). Further research (Koda et al., 2011) on the cholinergic mechanism showed that scopolamine (a muscarinic antagonist) and telenzepine (a preferential M_1_ MR antagonist that also binds M_2_ MRs) blocked galantamine-induced improvements in social isolation-induced prepulse inhibition deficits (Haddad et al., 1994). The activation of MRs by oxotremorine (a nonspecific muscarinic agonist) or N-desmethylclozapine (a specific M_1_ MR allosteric modulator) improved social isolation-induced prepulse inhibition deficits. Social isolation reduced the locomotor-suppressive response to oxotremorine (a nonselective MR agonist). Isolation also attenuated the N-desmethylclozapine-induced increase in prefrontal DA release. N-desmethylclozapine is a potent 5-HT_2_ receptor antagonist and ligand for the cloned 5-HT_6_ and 5-HT_7_ receptors. N-desmethylclozapine was used as an allosteric potentiator of M_1_ MR, but the abovementioned effects on 5-HT receptors should also be considered. Ago et al. (2011) attributed the lack of an effect of donepezil (vs. positive galantamine) on prepulse inhibition to the ability of donepezil, but not galantamine, to block the carbachol-induced increase in intracellular Ca^2+^ levels and attenuate the N-desmethylclozapine-induced increase in DA release in the mouse cerebral cortex. These results suggest that donepezil, but not galantamine, at least partially blocks MR function.

As mentioned above (Higashino et al., 2016), rivastigmine (a cholinesterase inhibitor) improved prepulse inhibition deficits, and this improvement was antagonized by telenzepine (a preferential M_1_ MR antagonist that also binds the M_2_ MR) but not by the nonselective NR antagonist mecamylamine. Rivastigmine increased extracellular ACh levels less than galantamine. Rivastigmine enhanced the effect of the M_1_ MR allosteric agonist N-desmethylclozapine on prefrontal DA release, and this increase was blocked by telenzepine.

As mentioned above (Ouchi et al., 2013), drugs used for ADHD therapy improved social isolation-induced latent learning deficits, and nicotinic antagonists were also reversible with the cholinergic muscarinic antagonist scopolamine.

Tacrine (an AChE inhibitor and allosteric muscarinic agonist) had no effect on social affiliation deficits induced by social isolation (Okada et al., 2015). In contrast, tacrine improved social isolation-induced deficits in fear memories, and this effect was reversed by scopolamine (an MR antagonist). Social isolation also downregulated the amount of calmodulin-dependent kinase II (p-CaMKII), cyclic AMP-responsive element binding protein (p-CREB) protein, and early growth response protein-1 (Egr-1), in the hippocampus, and tacrine attenuated these decreases, which were reversed by scopolamine.

Another aspect of social isolation on the cholinergic system was shown in autonomic control of heart rhythm. Prairie voles isolated for 4 weeks (vs paired) exhibited significantly elevated heart rate and reduced heart rate variability (Grippo et al., 2018). Administration of the β-adrenergic antagonist atenolol led to exaggerated reductions in heart rate, standard deviation of normal-to-normal intervals, and a lower amplitude of respiratory sinus arrhythmia in the isolated group vs paired. The administration of atropine (a muscarinic and nicotinic antagonist) led to an attenuated increase in heart rate in the isolated group vs paired and similar near-zero levels of respiratory sinus arrhythmia amplitude in both groups. During the tail suspension test, isolated vs paired animals exhibited significantly greater immobility. Atenolol administration did not influence immobility in paired animals. Atropine administration increased the duration of immobility vs vehicle. Atenolol administration increased the duration of immobility in isolated animals, but atropine did not influence immobility duration vs vehicle.

Social isolation (Koda et al., 2011) reduced the locomotor-suppressive response to oxotremorine (a nonselective MR agonist).

### Social Isolation in Complex Conditions

Some specific conditions (epileptic seizures, Alzheimer’s disease, and anhedonia) are tightly associated with the cholinergic system in which social isolation changes the outcome. For example, social isolation in genetically determined symptoms of an Alzheimer’s disease mouse model (APP/PS1 double mutant, i.e., amyloid precursor protein/presenilin 1) prolonged latency in the working memory test (Huang et al., 2011), increased the Aβ42/Aβ40 ratio in the hippocampus, increased the number of manganese-superoxide-dismutase-positive neurons in the CA1, CA2, and CA3 hippocampal areas, amygdala, and locus coeruleus, and decreased the number of ChAT-positive neurons in the vertical and horizontal diagonal bands of Broca (Huang et al., 2011). However, the ratio of cholinergic neurons (expressing VAChT) to the total number of activated neurons (cFos positive) was not changed in isolated rats after sleep deprivation (Deurveilher et al., 2013). Social isolation induced anhedonia and increased anxiety and biological markers of stress (Manouze et al., 2019). Group-housed mice treated with pilocarpine (a muscarinic agonist that was used to induce epileptic seizures) showed increased levels of anhedonia, anxiety and stress markers and decreased cognitive performance compared to the control group. Anxiety correlated linearly with cognitive performance and stress markers independent of the experimental conditions. Pilocarpine-induced epileptic seizures were sixteen times more frequent in singly housed animals than animals kept in pairs. Daily interactions with an experimenter in otherwise singly housed animals were sufficient to produce results identical to animals kept in pairs.

Notably, two aspects may affect the influence of social isolation, seasonal variation, i.e., biological rhythm, and sex, i.e., the role of sex hormones. The effects of social isolation exhibited seasonal variations (Davanzo et al., 1966), with troughs in the summer. Another generally ignored aspect is the that rodents are nocturnal animals. Therefore, it is more natural to perform tests during the dark period, which is the active part of the day. However, only some papers refer to tests performed in the dark (Ouchi et al., 2013), which is a better experimental condition choice than tests performed in the light, i.e., during the inactive period of rodents. The time of tests is not mentioned in many reports.

The issue of sex differences is not clear. Some authors have found sex differences in isolation effects (Mccormick et al., 2005; Caruso et al., 2018). However, some authors have not found sex differences (Kling et al., 1969). A review article (Matsumoto et al., 2019) discussed the presence or absence of sex differences associated with social isolation.

## Discussion

**FIGURE 2:**
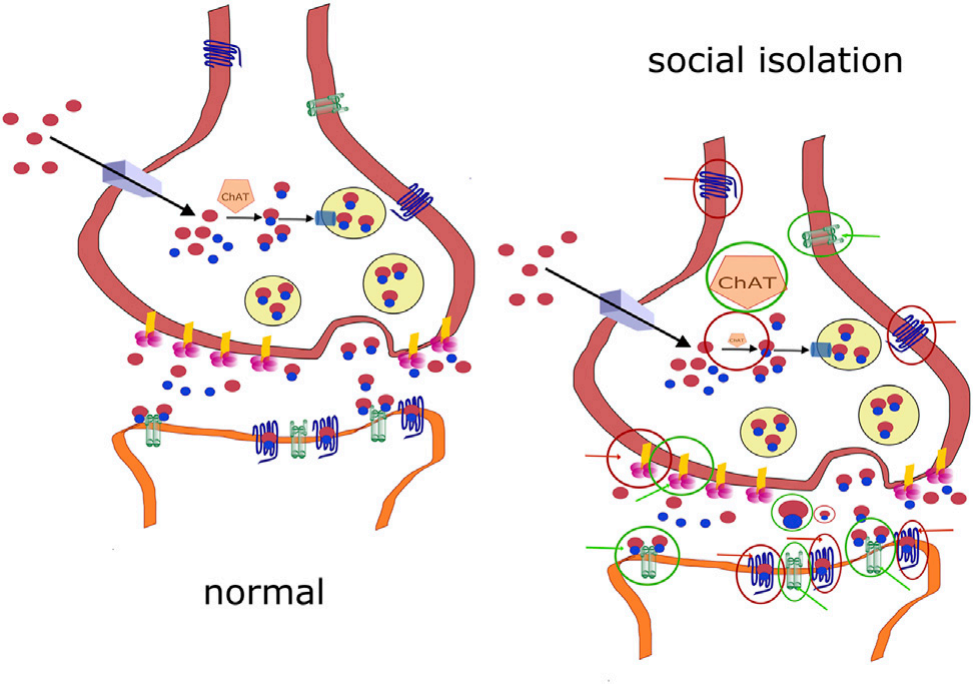
Comparison of normal signaling and changes in socially isolated animals. **(A)**: normal cholinergic signaling. **(B)**: changes in molecules involved in cholinergic signaling in socially isolated animals. A decrease is shown as a diminished symbol in the red circle, and an increase is shown as an enlarged symbol in the green circle (e.g., ChAT). Positive effects targeting the structure are shown as green circles with green arrows. Positive effects targeting the structure are shown as red circles with red arrows. If there is no change in the activity/amount/activation, then the molecule is shown under normal conditions.

Changes in molecules involved in cholinergic signaling elicited by social isolation are schematized in Figure 2. To summarize the role of the cholinergic system in social isolation, no changes were found in ChAT activity in the hippocampus or the prefrontal cortex. Decreases were found in cortical regions (unfortunately homogenized together: forebrain, temporal, parietal, and occipital cortices) and subcortical regions (homogenized together: hippocampus, globus pallidus, thalamus, nucleus basalis of Meynert, caudate nucleus, putamen, and striatum). An increase in ChAT activity was found in the nucleus accumbens. Decreased ACh concentrations were found in the whole infantile brain. The ACh content was increased in the diencephalon. No changes were found in the number of high-affinity choline transporters in the hippocampus.

AChE activity was not changed due to social isolation (AChE activity was unchanged in the cortex, prefrontal cortex, striatum, amygdala, diencephalon, brainstem, hypothalamus, midbrain, hippocampus, olfactory bulbs, pons plus medulla oblongata, and hippocampus).

However, decreased AChE activity was found in other cortical regions (forebrain, temporal, parietal, and occipital cortices) and subcortical regions (hippocampus, globus pallidus, thalamus, nucleus basalis of Meynert, caudate nucleus, putamen, and striatum) in rats. However, this finding has limited validity because this author homogenized all of these brain areas together.

AChE inhibition, which increases the amount of ACh, has different effects on social isolation. Neonatal treatment with pyridostigmine reduced the number of hippocampal CA1 and striatal synapses. However, the number of synapses was increased in isolated and isolated + pyridostigmine-treated rats at the age of 6 months. Other morphological changes were also found. Galantamine and rivastigmine, but not donepezil, improved prepulse inhibition deficits. Tacrine therapy had no effect on social isolation-induced social affiliation deficits. AChE inhibition produced no general outcome on social isolation-elucidated effects.

Antagonists with major nicotinic efficacy (see Table 2 for other receptors/enzyme targets) suppressed isolation-induced fighting behavior. The nicotinic system generally inhibits aggressive behavior. Rats kept in isolation showed increased sensitivity of striatal neurons to ACh. Nicotine attenuated separation-induced distress vocalizations in chicks. Arecoline and carbachol potentiated isolation-induced aggression in mice, and atropine suppressed aggressive behavior. Carbachol increased the firing rate of CA1 hippocampal neurons, with only a portion showing a dose-response curve. Isolation rearing increased the sensitivity of neurons, which showed a concentration-related increase in firing in response to carbachol, but had no effect on other neurons. Galantamine attenuated prepulse inhibition deficits elicited by isolation rearing. However, the galantamine-induced improvements in prepulse inhibition deficits were not prevented by the nicotinic ACh receptor antagonists mecamylamine and methyllycaconitine in apomorphine and MK-801 prepulse inhibition deficits. These data suggest that the complex interaction between receptor systems in social deficits induces effects on prepulse inhibition. Mecamylamine improved social isolation-induced latent learning deficits in water-finding conditions.

The brain muscarinic system initiates aggression. The effect of salbutamol (β_2_-adrenoceptor agonist) on oxotremorine-induced hypothermia was greater in isolated mice than group-reared mice. Scopolamine increased the frequency of separation-induced distress vocalizations. Isolation increased the marking scent behavior, which was suppressed by scopolamine. Arecoline, bethanechol, carbachol, oxotremorine, and pilocarpine potentiated isolation-induced aggression in mice, and atropine and scopolamine suppressed aggressive behavior. Impaired place and reversal learning are a consequence of isolation rearing, and scopolamine impairs both types of learning. An increase in affinity and decrease in the number of MRs in isolated rats were reported in cortical regions (forebrain, temporal, parietal, and occipital cortices) and subcortical regions (hippocampus, globus pallidus, thalamus, nucleus basalis of Meynert, caudate nucleus, putamen, and striatum). Separation stress, according to one paper, increased hippocampal M_1_ MR mRNA and MR density. However, another study did not find changes in hippocampal M_1_ mRNA expression. Carbachol increased the hippocampal neuron firing rate, as discussed above. The muscarinic antagonist scopolamine increased the number of ultrasonic vocalizations during short isolation. Galantamine attenuated and rivastigmine improved prepulse inhibition deficits (see also above), which were blocked by scopolamine and telenzepine. Oxotremorine and N-desmethylclozapine improved social isolation-induced prepulse inhibition deficits. Social isolation reduced the locomotor-suppressive response to oxotremorine. Drugs used for ADHD therapy improved social isolation-induced latent learning deficits, which were reversible with scopolamine. Tacrine improved social isolation-induced deficits in fear memories, and this effect was reversed by scopolamine. Social isolation reduced the locomotor-suppressive response to oxotremorine.

Isolation rearing also increased cholinergic fiber densities in the prefrontal cortices of the left hemisphere and the entorhinal cortex of the right hemisphere.

One important finding is that the orthosteric muscarinic binding site has a similar sequence within MR subtypes (see Table 2). This similarity makes the identification of specific subtypes involved in social isolation difficult. Some ligands also bind to off-target sites. Therefore, these drugs affect cholinergic and other neurotransmitter targets when administered systemically. Nicotinic drugs also bind to other targets. Table 2 shows that drugs used in isolation experiments bind to all MR subtypes with similar affinity, and some conclusions on the subtypes involved in these events are questionable. Drugs acting on NRs have similar properties. These drugs bind multiple subunits, and fundamental conclusions about the specific roles of NR subunits are also problematic. The table shows that AChE inhibitors affect not only AChE but also MRs and NRs regardless of other off-target sites (e.g., receptors, enzymes, and transporters).

To determine the specific MR subtype, it is necessary to consider the number of MR subtypes present in the specific tissue. Social isolation requires evaluation of the MR subtype number in the CNS, but it may also be necessary to determine the peripheral subtype number. There are two pioneering works using knockout animals determined the MR subtype number in peripheral tissues, such as the salivary glands, lung, heart, stomach, pancreas, bladder, and prostate (Ito et al., 2009), or in specific areas in the CNS (Oki et al., 2005) that determine the MR subtype numbers in the cerebral cortex, corpus striatum, hippocampus, hypothalamus, thalamus, midbrain, pons-medulla, cerebellum and spinal cord. Champtiaux and Changeux (2004) reviewed original studies on NR subtype/subunit presence in specific CNS areas. Both studies on MR subtypes came from the same laboratory, and there is one pitfall in these studies. The total amount of all MR subtypes, as determined by these studies in specific knockout (i.e., the sum of the density in M_1_+M_2_+M_3_+M_4_+M_5_), exceeded the density of all receptor subtypes determined in WT animals. This difference may be due to interactions between MR subtypes. However, it is suitable to consider what subtype is present in the respective CNS area. There are four MR subtypes (M_1_−M_4_) present in the cerebral cortex, corpus striatum, and thalamus (Oki et al., 2005). Three subtypes are present in the hippocampus (M_1_−M_3_) and pons medulla (M_2_−M_4_), two subtypes are present in the hypothalamus and midbrain (M_2_−M_3_), and only M_2_ is present in the cerebellum and spinal cord. Data from more specific brain areas involved in social isolation processes are not available.

**FIGURE 3:**
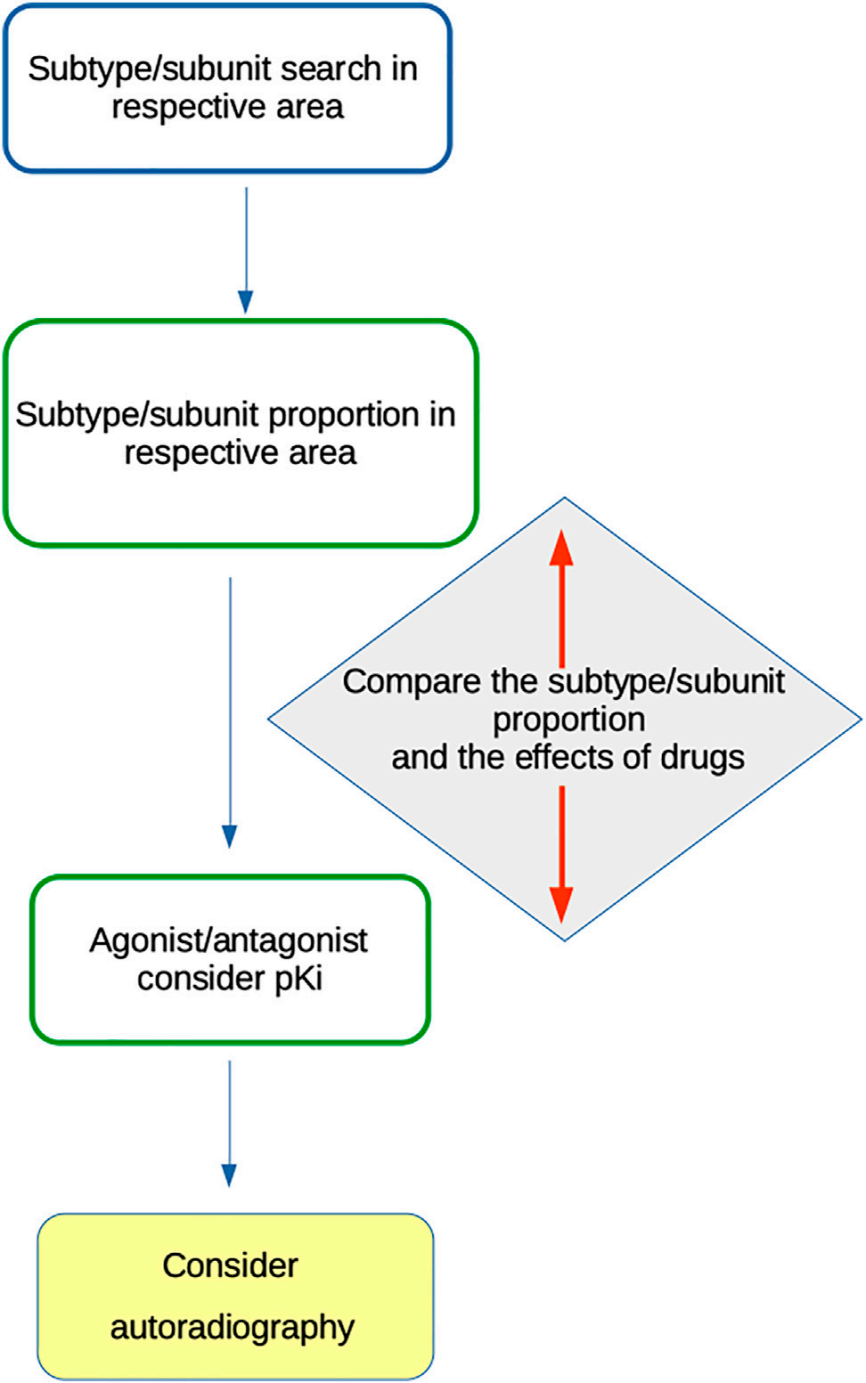
Algorithm for MR subtype or NR subunit role in social isolations.

Based on the above-mentioned facts, we suggest algorithms (Figure 3) for MR subtype or NR subunit roles in social isolations. All available data should be treated cautiously to avoid misinterpretation.

The following algorithm should be used for MR subtype determination:

1) Search for receptor subtype(s) present in the CNS area of interest (e.g., (Oki et al., 2005; Valuskova et al., 2018)).
2) Consider the proportion of specific subtypes, e.g., see the data in (Oki et al., 2005) or (Valuskova et al., 2018). Special attention should be given to knockout studies, which provide information about the number of receptors of specific subtypes.
3) Use the “specific” agonist and/or antagonist with respect to its pKi (see (Pascuzzo et al., 1984; Cantí et al., 1998; Becerra et al., 2001; Lockhart et al., 2001; Samochocki et al., 2003; Harada et al., 2010; Alexander et al., 2017a; Alexander et al., 2017b; Myslivecek, 2019) and the IUPHAR/BPS Guide to Pharmacology (www.guidetophamacology.org)).
4) Compare the subtype proportion and the effects of drugs.
5) Consider autoradiography as a method to verify the presence of specific subtypes in the respective CNS area. For example, M_1_ vs other MR subtypes may be determined using a specific protocol by Valuskova et al. (2018), which was verified in knockout animals.

The algorithm for NR subunit determination should be similar with dissimilarities, such as subtype vs. subunit presence, presence of a specific NR subtype composed of specific subunits, specific drugs with agonistic, antagonistic action, etc.

For both types of receptors, i.e., MRs and NRs, the off-target sites (receptors, enzymes, and transporters) should also be considered. A similar result is true for AChE inhibition. Table 2 illustrates the heterogenous nature of targets of different AChE inhibitors.

Another aspect that may contribute to the social isolation effects on cholinergic neurons may be the neurotransmitter switch, which is connected with the neurotransmitter receptor switch. Although a correlation between switching and receptor matching was repeatedly observed, how one influences the other is not understood (Li et al., 2020).

## Conclusion

The role of the cholinergic system in social isolation is indisputable, but it is difficult to determine the specific pathways involved in this role. Although the role of the cholinergic system in social isolation has been demonstrated repeatedly, the conclusion is not as evident as it should be. The first obstacle in the determination of social isolation effects is the variety of experimental conditions, as documented in Table 1. The isolation period varies greatly (from 1 h to 165 days), and the beginning of the isolation also differs (from birth to adulthood). Another aspect of the effects of social isolation on cholinergic signaling that is generally ignored is the fact that cholinergic drugs affect multiple targets.

As reviewed here, the changes in ACh content, ChAT activity, and AChE activity are discrete and limited to specific areas. The ACh content was increased in the diencephalon via isolation. An increase in ChAT activity was found in the nucleus accumbens, and decreases were found in cortical regions. The inhibition of AChE had various effects. Some inhibitors improved social isolation-induced deficits, and other inhibitors did not improve these deficits. Therefore, the receptors are main targets in social isolation. The blockade of NRs generally attenuates social isolation-induced behavior/vocalization. The activation of these receptors has opposite effects. Compared to NRs, the activation of MRs increased isolation-induced effects. MR blockade suppressed these effects. Social isolation also decreased the number of MRs. However, we cannot expect that receptors change their function *per se*. It is plausible to expect that discrete changes in cholinergic signaling and other neurotransmitter systems led to the different roles of NRs and MRs in social isolation studies.

As reviewed here, conclusions that a specific MR or NR subtype is involved should be drawn cautiously because the drugs used in these studies lack sufficient specificity for MR or NR subtypes. To determine the role of a specific receptor subtype, the presence of a specific subtype in the respective CNS area should be considered, as determined in studies with the absence of a specific receptor subtype and careful application of less or more specific agonists/antagonists (see Figure 3). Similarly, one should be cautious with AChE inhibition.
